# Chronic Venous Disease during Pregnancy Is Related to Inflammation of the Umbilical Cord: Role of Allograft Inflammatory Factor 1 (AIF-1) and Interleukins 10 (IL-10), IL-12 and IL-18

**DOI:** 10.3390/jpm13060956

**Published:** 2023-06-05

**Authors:** Lara Sánchez-Trujillo, Oscar Fraile-Martinez, Cielo García-Montero, Luis M. García-Puente, Luis G. Guijarro, Diego De Leon-Oliva, Diego Liviu Boaru, David Gardón-Alburquerque, María del Val Toledo Lobo, Mar Royuela, Ignacio García-Tuñón, Antonio Rios-Parra, Juan A. De León-Luis, Coral Bravo, Melchor Álvarez-Mon, Julia Bujan, Miguel A. Saez, Natalio García-Honduvilla, Miguel A. Ortega

**Affiliations:** 1 Department of Medicine and Medical Specialities, Faculty of Medicine and Health Sciences, University of Alcalá, 28801 Alcalá de Henares, Spain; larasancheztrujillo@gmail.com (L.S.-T.); oscarfra.7@hotmail.com (O.F.-M.); cielo.gmontero@gmail.com (C.G.-M.); lmgarciapuente@gmail.com (L.M.G.-P.); luis.gonzalez@uah.es (L.G.G.); diegodleonoliva01@gmail.com (D.D.L.-O.); diego.boaru@edu.uah.es (D.L.B.); david.gardon@edu.uah.es (D.G.-A.); mademons@gmail.com (M.Á.-M.); mjulia.bujan@uah.es (J.B.); msaega1@oc.mde.es (M.A.S.); natalio.garcia@uah.es (N.G.-H.); 2 Ramón y Cajal Institute of Sanitary Research (IRYCIS), 28034 Madrid, Spain; mval.toledo@uah.es (M.d.V.T.L.); riosparraanto@yahoo.es (A.R.-P.); 3 Deparment of Pediatrics, Hospital Universitario Principe de Asturias, 28801 Alcalá de Henares, Spain; 4 Department of Systems Biology, Faculty of Medicine and Health Sciences (Networking Research Center on for Liver and Digestive Diseases (CIBEREHD)), University of Alcalá, 28801 Alcalá de Henares, Spain; 5 Department of Biomedicine and Biotechnology, University of Alcalá, 28801 Alcalá de Henares, Spain; mar.royuela@uah.es (M.R.); ignacio.tunon@uah.es (I.G.-T.); 6 Pathological Anatomy Service, University Hospital Príncipe de Asturias, 28806 Alcalá de Henares, Spain; 7 Department of Public and Maternal and Child Health, School of Medicine, Complutense University of Madrid, 28040 Madrid, Spain; jaleon@ucm.es (J.A.D.L.-L.); cbravoarribas@gmail.com (C.B.); 8 Department of Obstetrics and Gynecology, University Hospital Gregorio Marañón, 28009 Madrid, Spain; 9 Health Research Institute Gregorio Marañón, 28009 Madrid, Spain; 10 Immune System Diseases-Rheumatology and Internal Medicine Service, University Hospital Príncipe de Asturias, CIBEREHD, 28806 Alcalá de Henares, Spain; 11 Pathological Anatomy Service, Central University Hospital of Defence-UAH Madrid, 28801 Alcala de Henares, Spain

**Keywords:** chronic venous disease (CVD), pregnancy, umbilical cord, inflammation, Allograft inflammatory factor 1 (AIF-1), Interleukin 10 (IL-10), IL-12A, IL-18

## Abstract

Chronic venous disease (CVD) is a common condition that affects the veins in the lower limbs, resulting in a variety of symptoms, such as swelling, pain, and varicose veins (VVs). The plenty hormonal, hemodynamic and mechanical changes occurred in pregnancy make women especially vulnerable to suffer from this condition in this period. Previous works have identified that CVD is associated with an increased inflammatory milieu and significant damage in maternofetal tissues, such as the umbilical cord. However, the inflammatory status of this structure in these patients has not been studied yet. Thus, the aim of the present study was to examine gene and protein expression of a set of inflammatory markers—Allograft inflammatory factor 1 (AIF-1), the proinflammatory cytokines interleukin 12A (IL-12A) and IL-18 and the anti-inflammatory product IL-10—in the umbilical cord of women with CVD during pregnancy (N = 62) and healthy pregnant women (HC; N = 52) by the use of real time qPCR and immunohistochemistry (IHC). Our results demonstrate that the umbilical cord tissue from CVD women exhibit an increased expression of AIF-1, IL-12A and IL-18 along with a decrease in IL-10. Therefore, our study suggests an inflammatory status of this structure related to CVD. Further studies should be conducted to evaluate the expression of other inflammatory markers, as well as to analyze the maternofetal impact of these findings.

## 1. Introduction

Chronic venous disease (CVD) is a relatively common vascular disorder that causes an increase in venous pressure and is characterized by a lack of venous return to the lower extremities [1,2]. The clinical symptoms of CVD range widely, from minor changes in the venous system, such as reticular veins, telangiectasia, or varicose veins (VVs), to severe manifestations designed as chronic venous insufficiency (CVI), which can be present in the form of edema, skin changes, such as lipodermatosclerosis, and active ulcerations [3,4]. Pregnancy is one of the main risk factors for the development of CVD [5]. According to estimates, up to 40% of women experience this disease during pregnancy, increasing their chance of suffering from this condition again in subsequent pregnancies. [6]. This increased risk of CVD in pregnancy is explained by the hemodynamic, mechanic and hormonal changes that occur during pregnancy, including fetal compression of the iliac veins, vasodilatation, and secondary stasis with reduced flow velocity and valvular incompetence [7,8,9,10,11]. However, CVD pathogenesis is not completely understood, and further research is needed to fully decipher its effects on fetuses and newborns.

Pregnancy is a proinflammatory state, in which cytokines mediate signaling that directs the biological processes that constitute pregnancy from implantation to delivery [12]. The cytokine profile changes over the course of pregnancy; thus, maintaining a proper ratio of proinflammatory to anti-inflammatory cytokines is essential for the appropriate development of pregnancy [13]. Different obstetric conditions can alter the cytochemical balance, and among them, CVD also seems to produce a switch into a proinflammatory cytokine profiling in both pregnant women and their newborns [14]. Although this pro-inflammatory environment seems to be determinant for the future child, future research is needed to determine its impact on the fetus and the newborn.

The umbilical cord is an anatomical structure that connects the fetus and the placenta supplying adequate nutrition, oxygenation, and proper waste disposal. Vascular alterations of the umbilical cord can compromise or modify fetal blood flow, which may imply fetal compromise, increased perinatal and neonatal morbidity and mortality [15]. Moreover, alterations at the level of the umbilical cord are closely related to fetal programming, and thus, can impact the health of the newborn at birth and beyond [16,17]. Previous research has demonstrated how CVD causes placental changes that result in cellular harm, hypoxia, increased calcification, oxidative stress, and increased vascularization [18,19,20,21], whereas evidence of increased hypoxia and oxidative stress has also been demonstrated in the umbilical cord of women with pregnancy-associated CVD [22]. In addition, previous studies have found significant alterations in inflammatory biomarkers associated with different pathologies in pregnancy, such as pre-eclampsia and other complications [23,24,25]; however, to date, there are no studies evaluating the immunoinflammatory status of the umbilical cord from women with CVD. Allograft inflammatory factor 1 (AIF-1) is a product importantly implicated in several cellular events and its expression is induced by different proinflammatory cytokines [26]. The pathogenic role of AIF-1 has been proven in several inflammatory, vascular and systemic disorders [27], although little is known regarding its role in the umbilical cord under pregnancy complications. Likewise, the role of inflammatory cytokines in the umbilical cord of these patients also warranted further efforts.

Therefore, the aim of the present work is to define a possible role of AIF-1 and the inflammatory environment in the umbilical cord of women with CVD during pregnancy. Having this purpose, the umbilical cord tissue from pregnant women with CVD will be used to examine gene and protein expression of AIF-1, interleukin 10 (IL-10), IL-12A, and IL-18 by real-time PCR (RT-qPCR) and immunohistochemistry, respectively.

## 2. Patients and Methods

### 2.1. Study Design

In the current investigation, 114 pregnant women in their third trimester participated in an observational, analytical, prospective study. In total, 52 women without a history of CVD (referred to as healthy control, HC) and 62 women with a clinical diagnosis of CVD, according to the CEAP classification [28], were included. The interquartile range (IQR) for the CVD group was 22–40 years, while the median age for the HC group was 34 (IQR, 27–41 years). For women with CVD, the median gestational period was 40.5 weeks (with an IQR of 39–41.5 weeks), whereas for women with HC, it was 41 weeks (IQR, 39–42 weeks).

Each patient received the necessary information before enrollment, giving their signed, written consent. This work was carried out in accordance with the ethical principles of autonomy, beneficence, non-maleficence, and distributive justice, as well as the rules of good clinical practice, the principles of the Declaration of Helsinki (2013), and the Oviedo Convention, and it was approved by the Clinical Research Ethics Committee of the Central University Hospital of Defense University of Alcalá (37/17) (1997).

### 2.2. Inclusion/Exclusion Criteria and Clinical Assessment

In this study, we included pregnant women aged >18 years old, with or without clinical presentation of CVD in their lower limbs during the third trimester. Pregnant women were excluded from this study if they presented: (1) Endocrine disorders (i.e., diabetes mellitus); (2) high blood pressure (HBP); (3) a body mass index (BMI) ≥ 25 kg/m^2^; (4) Unhealthy or toxicologic habits; (5) Active infectious or autoimmune diseases; (6) venous malformations; (7) kidney, heart, or lung failure; (8) preeclampsia; (9) elevated liver enzymes and low platelets (HELLP syndrome); (10) intrauterine growth restriction with a known cause; (11) pathological lesions in the placenta or umbilical cord; and (12) Prior evidence of CVD. In this trial, acetylsalicylic acid was not used to treat or prevent preeclampsia in elder pregnant women.

Each woman’s clinical history was updated during the third trimester appointment, and physical tests were completed. Eco-Doppler (Portable M-Turbo Eco-Doppler; SonoSite, Inc., Bothell, WA, USA) operating at 7.5 MHz was used to analyze ultrasound images of the lower limbs.

Regarding the sociodemographic features of the population under study, there were no discernible differences between women with CVD and those without it in terms of the number of pregnancies, which was 33 (53.2%) for those with CVD and 19 (36.5%) for those without it (Table 1). Additionally, there were no statistically significant differences between the two groups in terms of their clinical characteristics (gestational age, C-section delivery, prior pregnancies and abortions, regularity of menstrual cycles, and sedentary occupation) (Table 1).

### 2.3. Tissue Samples Management

All 114 patients provided postpartum umbilical cord samples. After the placenta was expelled, umbilical tissue biopsies were taken and used for immunohistochemical, genetic, and molecular research. They were then divided into two sterile tubes, one of which contained minimal essential medium (MEM; Thermo Fisher Scientific, Inc., Waltham, MA, USA) with 1% antibiotic/antimycotic (streptomycin, amphotericin B, and penicillin; Thermo Fisher Scientific, Inc.), and the (Ambion; Thermo Fisher Scientific, Inc., Waltham, MA, USA). The samples were then processed in a sterile environment in a class II laminar flow hood (Telstar AV 30/70 Müller 220 V 50 MHz; Telstar; Azbil Corporation Marunouchi, Chiyoda-ku, Tokyo, Japan). After that, materials were kept for subsequent processing for gene expression research in 1 mL of RNAlater^®^ at −80 °C.

Erythrocytes were removed by washing and rehydrating preserved MEM samples five times in antibiotic-free MEM. Following that, they were cut into 2 cm-long pieces using a scalpel and fixed in F13 (a solution made up of 60% ethanol, 20% methanol, 7% polyethylene glycol, and 13% distilled water), in accordance with protocols that have been gathered [20]. Molds were then used to create samples with embedded paraffin. Thermo Fisher Scientific, Inc., Waltham, MA, USA, used an HM 350 S rotary microtome to cut slices that were 5 µm thick after the paraffin had set. They were then collected on glass slides that had been coated with 10% polylysine to aid in the sections’ adhesion after being stretched in a hot water bath.

### 2.4. Gene Expression Investigations Utilizing Quantitative PCR and Reverse Transcription (RT-qPCR)

We first extracted RNA using the guanidinium thiocyanate-phenol-chloroform technique. The mRNA expression levels of particular genes can be studied using this technique.

Reverse transcription (RT) was used to create complementary DNA (cDNA) from 50 ng/µL of RNA samples. Each sample was combined with 4 µL of 0.25 µg/µL oligo-dT solution (Thermo Fisher Scientific, Inc., Waltham, MA, USA) and then placed in an AccuBlock dry bath (Labnet International Inc., Edison, NJ, USA) at 65 °C for 10 min to cause RNA denaturation. Following that, 10 µL of a reverse transcription mix containing the following items was added to the samples on ice. The following ingredients are from Thermo Fisher Scientific, Inc.: 2.8 µL of First Strand Buffer 5× (250 mM Tris-HCl at pH 8.3; 375 mM KCl:15 mM MgCl_2_); 1 µL of RT enzyme; 2 µL of 10 mM deoxyribonucleotide triphosphate; 2 µL of 0.1 M dithiothreitol; 1.7 µL of DNase and RNase free water; 0.5 µL of RNase inhibitor (RNase Out).

Using a G-Storm GS1 thermocycler, reverse transcription was carried out (G-Storm Ltd., Middlesbrough, UK). In order to promote cDNA synthesis, the samples were subsequently incubated at 37 °C for one hour and fifteen minutes. After that, the temperature was raised to 70 °C and maintained there for 15 min, denaturing the reverse transcriptase. The temperature was then gradually decreased to 4 °C. In RNA samples, where the M-MLV RT enzyme was substituted with DNase- and RNase-free water, negative reverse transcription was likewise carried out to guarantee the lack of genomic DNA contamination. cDNA made at room temperature was diluted 1:20 in water devoid of DNase and RNase and kept at −20 °C until needed.

Via the Primer-BLAST and AutoDimer online programs, specific primers for the chosen genes (Table 2) were created from scratch. To standardize the findings, the constitutively expressed TATA-box binding protein (TBP) gene was employed as a control. The proportional amounts of mRNA were used to express the gene expression units. The relative standard curve method was used to conduct RT-qPCR using a StepOnePlus^TM^ System (Applied Biosystems; Thermo Fisher Scientific, Inc.). The following was the outcome of the reaction: 5 µL of sample—mixed at 1:20 with 10 µL iQ^™^ SYBR^®^ Green Supermix (Bio-Rad Laboratories, Hercules, CA, USA)—was mixed with 1 µL for each forward and reverse primers, and 3 µL of DNase and RNase-free water, which were then added to a MicroAmp^®^ 96-well plate (Applied Biosystems; Thermo Fisher Scientific, Inc., Waltham, MA, USA).

The following thermocycling conditions were used: initial denaturation for 10 min at 95 °C, denaturation for 15 s at 95 °C, annealing at different temperatures depending on the melting temperature of each primer pair for 30 s, and elongation at 72 °C for 1 min, for 40 to 45 cycles. Then, a dissociation curve was produced for 15 s at 95 °C, 1 min at 60 °C, 15 s at 95 °C, and 15 s at 60 °C. At the conclusion of each repeat cycle (amplification), as well as at other points throughout the dissociation curve, fluorescence was detected.

The information gathered from the aforementioned genes was included in a standard curve that was created through serial dilutions of a mixture of materials that were added to each plate in accordance with the constitutive expression of TBP (as per the manufacturer’s guidelines). All umbilical cord tissue samples underwent two rounds of this RT-qPCR.

### 2.5. Immunohistochemical Studies

The samples that were kept in MEM were sliced into pieces and kept in various fixatives, including F13 (60% ethanol, 20% methanol, 7% polyethylene glycol, and 13% distilled water), after being repeatedly washed and hydrated with antibiotic-free medium to remove blood cells. The samples were dried in accordance with established procedures after being fixed for the required amount of time in each fixing solution.

According to the following procedure, the antigen–antibody reaction was detected using the ABC (avidin-biotin complex) method with peroxidase or alkaline phosphatase as the chromogen: (1) incubation with the main antibody; (2) washing three times with 1 PBS for five minutes each; (3) blocking of non-specific binding sites with 3% bovine serum albumin (BSA) in PBS for thirty minutes at room temperature; (4) incubating the primary antibody (Table 3) diluted in 3% BSA and PBS for an overnight period at room temperature; (5) rinsing three times with PBS for five minutes each; (6) incubating the secondary antibody coupled with biotin and diluted in PBS for one hour and thirty minutes at room temperature; (7) rinsing three times with PBS for five minutes each; and (8) incubating the avidin-peroxidase conjugate ExtrAvidin^®^-Peroxidase (Sigma-Aldrich, St. Louis, MO, USA) (diluted 1/200 in PBS for 60 min at room temperature); (9) 3 PBS rinses, each lasting 5 min; (10) Development was performed using the DAB Kit, SK-4100, a diaminobenzidine chromogenic substrate (Vector Laboratories, Burlingame, CA, USA). The chromogenic substrate was made up of 5 mL of distilled water, 2 drops of buffer, 4 drops of DAB, and 2 drops of hydrogen peroxide just before exposure; this method leaves a dark stain. Furthermore, the following was conducted: (11) stopping the reaction by rinsing three times in distilled water for five minutes each; (12) staining the nuclei with Carazzi haematoxylin for five to fifteen minutes to get contrast; (13) washing in running water for ten minutes; and (14) mounting in aqueous medium with plasdone. Sections of the same tissue were used in all immunohistochemical experiments as a negative control, wherein blocking solution incubation took the place of the primary antibody’s incubation.

### 2.6. Statistical and Microscopical Determination

The IRS-Score method was conducted to evaluate five sections and ten fields for each patient in the designated groups [29]. The preparations were inspected using a Zeiss Axiophot optical microscope with an AxioCam HRc digital camera (Carl Zeiss, Jena, Germany).

The Mann–Whitney U test was used in the statistical study using the GraphPad Prism^®^ 6.0 (San Diego, CA, USA) program. When appropriate, we used Fisher’s exact or Pearson’s chi-squared tests if the research variables were not quantitative. In order to represent the statistics, the mean and interquartile range are used. The thresholds for significance were *p* = 0.05 (*), *p* = 0.01 (**), and *p* = 0.001 (***).

## 3. Results

### 3.1. The Umbilical Cord of Women with Chronic Venous Disease during Pregnancy Exhibit Increased Allograft Inflammatory Factor (AIF-1) Expression

**Figure 1:**
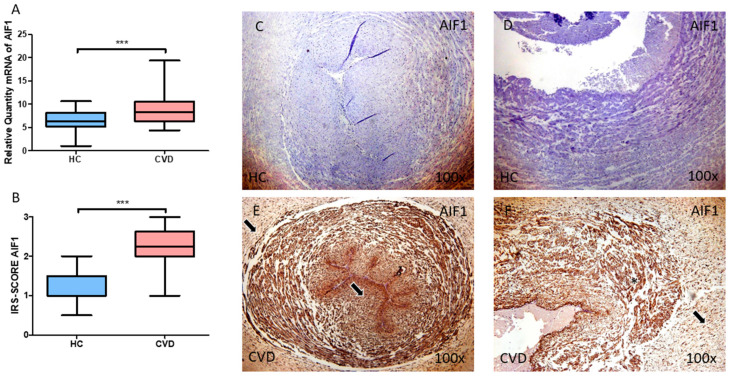
(**A**) mRNA expression levels for AIF-1 by RT-qPCR in the umbilical cord. (**B**) Levels of IRS-SCORE of AIF-1 protein expression in the umbilical cord. (**C**–**F**) Images showing the immunohistochemistry of AIF-1 in the umbilical cord. For both the umbilical artery (**C**,**E**) and umbilical vein (**D**,**F**), increased expression of AIF-1 is more marked in the tunica intima and media when compared to the aventitia (black arrows). CVD = Newborns of women diagnosed with chronic venous disease during pregnancy. HC = Control without venous pathology. *p* < 0.001 (***).

Firstly, gene and protein expression of *AIF-1* was studied in the umbilical cord of women with CVD. Our results report that there is a statistically significant increase in *AIF-1* gene expression in the umbilical cord tissue of pregnant women with CVD (Figure 1A; CVD = 9.001 ± 3.661, HC = 6.347 ± 2.170, *** *p* = 0.0002). Similarly, the umbilical cord of women with CVD displayed a significant augmentation in protein expression of AIF-1, according to histological analysis (Figure 1B; CVD = 2.226 ± 0.612, HC = 1.163 ± 0.512, *** *p* < 0.001). Regarding histopathological images, women with CVD had significantly more AIF-1 protein expression in both the umbilical artery and umbilical vein (Figure 1C–F).

### 3.2. The Umbilical Cord of Women with Chronic Venous Disease during Pregnancy Display a Greater Expression of the Proinflammatory Cytokines IL-12A and IL-18

**Figure 2:**
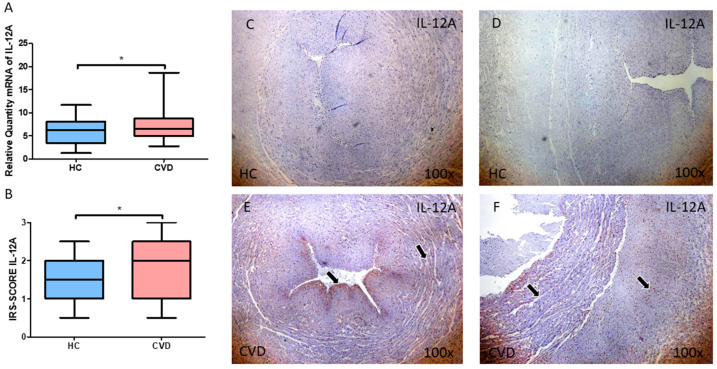
(**A**) mRNA expression levels for IL-12A by RT-qPCR in the umbilical cord. (**B**) Levels of IRS-SCORE of IL-12A protein expression in the umbilical cord. For both the umbilical artery (**C**,**E**) and umbilical vein (**D**,**F**), increased expression of IL-12 is more marked in the tunica intima and media when compared to the aventitia (black arrows). CVD = Newborns of women diagnosed with chronic venous disease during pregnancy. HC = Control without venous pathology. *p* = 0.05 (*).

We then explored the gene and protein expression of the proinflammatory cytokines IL-12A and IL-18 in the umbilical cord. When compared to HC, women with CVD had significantly increased levels of IL-12A gene expression in this tissue (Figure 2A; CVD = 7.502 ± 3.541, HC = 5.919 ± 2.840, * *p* = 0.0397). When the umbilical cord of women with CVD were subjected to a histological analysis, this significant raise was also defined (Figure 2B; CVD = 1.863 ± 0.720, HC = 1.596 ± 0.534, * *p* = 0.0341). Histological images show that the expression of IL-12A is higher in the CVD group in both the umbilical artery and umbilical vein when compared to HC (Figure 2C–F).

**Figure 3:**
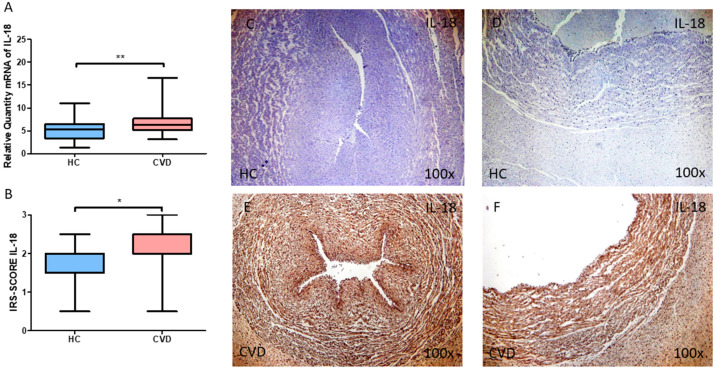
(**A**) mRNA expression levels for IL-18 by RT-qPCR in the umbilical cord. (**B**) Levels of IRS-SCORE of IL-18 protein expression in the umbilical cord. (**C**–**F**) Images showing the immunohistochemistry of IL-18 in the umbilical cord, including umbilical artery and vein. CVD = Newborns of women diagnosed with chronic venous disease during pregnancy. HC = Control without venous pathology. *p* = 0.05 (*), *p* = 0.01 (**).

Regarding IL-18, we observed that the gene expression of this marker in the umbilical cord was significantly increased in the CVD group when compared to HC (Figure 3A; CVD = 6.825 ± 2.535, HC = 5.349 ± 2.522, ** *p* = 0.0036). For protein expression, a significant increase of IL-18 was also observed in the CVD group (Figure 3B; CVD = 2.032 ± 0.658, HC = 1.798 ± 0.467, * *p* = 0.0231). Histologically, the increased protein expression of this marker was reported in in both the umbilical artery and umbilical vein of women with CVD (Figure 3C–F).

### 3.3. The Umbilical Cord of Women with Chronic Venous Disease during Pregnancy Show a Marked Reduction in the Expression of the Anti-Inflammatory Cytokine IL-10

**Figure 4:**
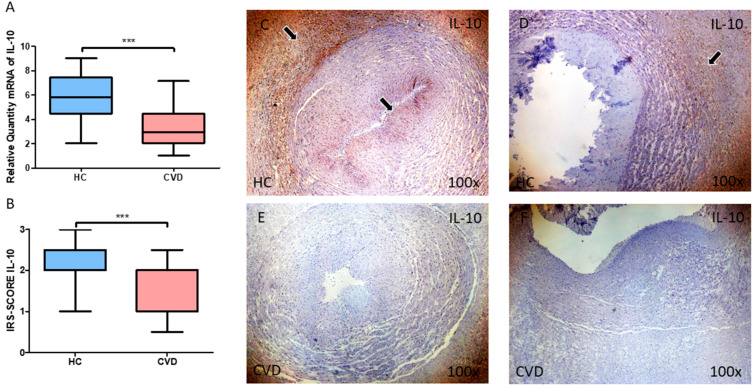
(**A**) mRNA expression levels for IL-10 by RT-qPCR in the umbilical cord. (**B**) Levels of IRS-SCORE of IL-10 protein expression in the umbilical cord. (**C**–**F**). As shown, for both the umbilical artery and vein, an increased IL-10 expression is observed in the perivascular Wharton’s jelly, although for the umbilical artery there is also a notable expression in the intima and media layer (**C**,**E**, black arrows) Images showing the immunohistochemistry of IL-10 in the umbilical cord. CVD = Newborns of women diagnosed with chronic venous disease during pregnancy. HC = Control without venous pathology. *p* < 0.001 (***).

Finally, we explored the gene and protein expression of the anti-inflammatory cytokine IL-10 in the umbilical cord of women with CVD. Our results show a decreased gene expression of this cytokine in this tissue in comparison to the HC (Figure 4A; CVD = 3.272 ± 1.500, HC = 5.800 ± 1.947, *** *p* < 0.001). In the case of protein expression, a significant decrease of IL-10 was also observed in the CVD group (Figure 4B; CVD = 1.339 ± 0.606, HC = 2.202 ± 0.628, *** *p* < 0.001). Histologically, the increased protein expression of this marker was reported in both the umbilical artery and umbilical vein of the HC, in comparison to those affected with CVD (Figure 4C,D).

## 4. Discussion

In this work, we demonstrated an increased gene and protein expression of AIF-1, IL-12A and IL-18 in the umbilical cords of pregnant women with CVD. Similarly, a significant decrease in the expression of IL-10 is also reported in these subjects. It is not clear whether inflammation is caused by CVD or instead, CVD is partly consequence of inflammation. Even, the different hemodynamical, hormonal and mechanical factors associated with CVD might also relate to inflammation, and both can be a consequence of them. However, it is more plausible that all these factors are connected, as according to previous evidence, the inflammatory response is essential in the onset and development of CVD, and in turn, increased levels of tissue and systemic proinflammatory markers are directly associated with CEAP severity [4,30]. Howsoever, we previously evidenced that CVD during pregnancy is associated with an increased proinflammatory status observed in the serum of the mother and newborns, as well as damage in maternofetal structures, such as the umbilical cord [14,31,32]. Furthermore, the pathogenic environment related to CVD can affect both the mother and the fetus [33,34], thus demonstrating the need of additional studies evaluating this complex association.

AIF-1 is a 17 kDa cytosolic protein that binds calcium and actin, acting as a scaffold/adaptor protein. The *AIF-1* gene is situated in the MHC class III region, close to the TNF-α, TNF-β, and NF-κB, complement cascade protein genes, and surface glycoprotein genes [27]. The relevance of AIF-1 in multiple diseases has been demonstrated broadly [35]. AIF-1 exert a critical immunomodulatory action, boosting the expression of many inflammatory mediators and favor macrophage and vascular smooth muscle cell proliferation and migration [36]. The role of AIF-1 in the umbilical cord has been poorly studied. Nevertheless, Jia et al. [37] demonstrated that the increased AIF-1 expression by human umbilical vein endothelial cells (HUVECs) enhanced the proliferation and migration of these cells, which could promote angiogenesis probably through augmenting the expression of basic fibroblast growth factor (bFGF). An increased angiogenesis has been reported in the placenta of women with CVD [38], and even though this process has not been studied in the umbilical cord yet, the increased expression of AIF-1 could be involved in this process, although further studies should be aimed to explore this relationship. In the same line, previous works have evidenced that AIF-1 can stimulate the expression of inducible nitric oxide synthase (iNOS) [39]. An enhanced iNOS expression has been previously identified in the umbilical cord of pregnant women with CVD [22], suggesting a potential pathogenic association between AIF-1 and iNOS in these patients.

Previous works have found that an inflammatory environment activates AIF-1 [40]. In turn, AIF-1 seems to enhance the production of several proinflammatory cytokines and chemokines [41]. We previously evidenced a significant upregulation of many proinflammatory cytokines in the sera and maternofetal tissues from pregnant women with CVD and their newborns, which could be tightly linked to AIF-1 upregulation [14,31]. In this study, we also observed an increase in the proinflammatory cytokine IL-12A in the plasma of newborns with CVD, along with a decrease in the anti-inflammatory cytokine IL-10 [14]. However, the tissue expression of these cytokines in the umbilical tissue of these cytokines had not been studied before. In our study, we observe the same results in the umbilical cord of women with CVD, along with an increase in the expression of IL-18.

IL-12 is a heterodimeric proinflammatory cytokine formed by IL-12A (p35) and IL-12B (p40) subunits, belonging to the IL-12 family together with IL-23, IL-17, and IL-35 [42]. IL-12 is produced by B cells, dendritic cells, and macrophages, and it is involved in interferon gamma (IFN-γ) production, T cell differentiation, and function [43], having also been related to pathogenic Th1 differentiation [44]. IL-12 is also associated with an imbalance in Th1/Th2 cells. This imbalance has been associated with pregnancy complications, such as recurrent spontaneous abortion, obstetric complications, and poor pregnancy outcomes [45]. The relevance of increased IL-12 levels had also observed similar alterations in pregnant women with severe pre-eclampsia [46]. It is likely that high levels of IL-12 could be associated with pathological conditions such as CVD, although here we have only evidenced an increased expression of IL-12A subunit. Thus, further works should corroborate our results and clarify the mechanisms involved in its dysregulation, and its maternal and fetal consequences.

IL-18 is a pro-inflammatory cytokine that belongs to the IL-1 family [47]. Originally, it was called the IFN-γ inducing factor, given its involvement in inducing the production of IFN-γ in Th1 lymphocytes without specifying the interaction with the TCR receptor [48]. The binding of IL-18 with its receptor is involved in the signaling and activation of the MAPK and NF-kB pathways [48]. Various types of immune and non-immune cells are able to express this cytokine [47,48]. In turn, its relationship with the type 2 immune response has also been demonstrated, by inducing the production of IL-13, being implicated in allergic diseases and in other processes, such as sepsis, inflammatory bowel disease, acute kidney injury and autoimmune diseases, i.e., juvenile idiomatic arthritis or systemic lupus erythematosus [47,49]. Likewise, IL-18 is related to the so-called inflammasome: a cytoplasmic multimeric protein complex which cleaves the inactive precursors of IL-1β and IL-18 into bioactive cytokines under inflammatory conditions [50]. Given its role in innate and acquired immunity, the increase in the expression of IL-18 at the level of the umbilical cord could have maternal–fetal implications. IL-18 is expressed at the maternal–fetal interface at the chorion and decidua levels [51] Increased expression at this level has been associated with an increased risk of preterm delivery [51], as well as recurrent spontaneous abortion [52]. Studies on pre-eclampsia, objectify an increase of IL-18 in serum and placenta compared to control healthy women [53], probably as part of the exacerbated inflammatory response that is part of the pathogenesis of preeclampsia. These changes in the expression of IL-18 in the umbilical cord of newborns with CVD, therefore, reflect an increase in the inflammatory response in CVD, although more studies are necessary to clarify its role and maternofetal repercussions.

Finally, we also demonstrated a decrease in the gene and protein expression of the anti-inflammatory IL-10. Additionally, in previous studies, we have reported decreased levels of anti-inflammatory cytokines IL-4, IL-10, and IL-13 in the serum of mothers with CVD and their newborns [14]. IL-10 is a cytokine with a decisive anti-inflammatory role [54,55]. It is secreted by different types of immune cells, mediating tolerance responses and regulatory T-cell responses [54,55] and it produces inhibition of IL-2 and interferon-gamma [56]. This cytokine exerts contrary effects to TNF-α. Previous studies have found that a concomitant reduction in IL-10 levels, with augmented TNF-α, might be related to pathological inflammation [57]. However, deficiencies in IL-10 have been associated with a plethora of pregnancy-related disorders, including infertility, spontaneous abortion, fetal growth restriction, preterm birth, preeclampsia, gestational hypertension [14,58] and also with CVD. Additionally, some studies have observed that decreased cord blood levels of IL-10 in newborns with a birth weight greater than the 95th percentile, born to healthy mothers without gestational pathology, establishes an inverse correlation between IL-10 levels and birth weight [59]. Additionally, some studies suggest that low levels of venous cord blood IL-10 are associated with an increased risk of suffering moderate or severe bronchopulmonary dysplasia in small, gestational age, preterm newborns [60]. The decreased expression of this cytokine in the umbilical cord of women with CVD is reflective of the inflammatory status associated with this condition in pregnancy. However, how this decrease affects the fetus and newborn in CVD remains to be clarified.

## 5. Conclusions

Inflammation is a common biological mechanism with essential functions for pregnancy development, although an exacerbated inflammatory milieu is a major characteristic of many obstetric complications. CVD is a condition associated with proinflammatory changes in pregnancy that affect maternofetal structures like the placenta and the umbilical cord. In this work, we have shown for the first time the existence of a significant increase in the gene and protein expression of AIF and cytokines IL-18 and IL-12, with a decrease of IL-10 in the umbilical cords of newborns whose mothers had gestational CVD. Alterations at the level of the umbilical cord are closely related to fetal programming, and thus, impact newborn health at birth and in later childhood. Further efforts are needed to analyze the maternal–fetal impact of these findings, in addition to the possible prognostic value of these markers, which may help in the early detection and diagnosis of CVD and its potential complications in the fetus and newborn.

## Figures and Tables

**Table 1:** Demographic and clinical features. Healthy control, or HC, stands for chronic venous disease.

	CVD (*n* = 62)	HC (*n* = 52)
Median age (IQR), years	33 (22–40)	34 (27–41)
Median gestational age (IQR), weeks	40.5 (39–41.5)	41 (39–42)
C-section delivery, *n* (%)	12 (19.4)	9 (17.3)
Vaginal delivery, *n* (%)	50 (80.6)	43 (82.7)
CVD (CEAP), *n* (%)		
CEAP 1	37 (59.7)	0 (0)
CEAP 2	21 (33.8)	0 (0)
CEAP 3	4 (6.5)	0 (0)
Previous pregnancies, *n* (%)	33 (53.2)	19 (36.5)
Previous abortions, *n* (%)	14 (22.6)	9 (17.3)
Regular menstrual cycles, *n* (%)	50 (80.6)	42 (80.7)
Sedentary profession, *n* (%)	41 (66.1)	40 (76.9)

**Table 2:** Primer sequences used in RT-qPCR and temperature (Tm).

Gene	Sequence Fwd (5′→3′)	Sequence Rev (5′→3′)	Temp
*TBP*	TGCACAGGAGCCAAGAGTGAA	CACATCACAGCTCCCCACCA	60 °C
*AIF-1*	TGAAAACCCTCCAGTCAGCG	GTCAGGGTAGCTGAACGTCT	60 °C
*IL-12A*	GCACAGTGGAGGCCTGTTTA	GCCAGGCAACTCCCATTAGT	60.2 °C
*IL-18*	GCTGAAGATGATGAAAACCTGGA	GAGGCCGATTTCCTTGGTCA	59.5 °C
*IL-10*	TGCTCTTGCAAAACCAAACCA	GGGAGGTCAGGGAAAACAGC	60 °C

**Table 3:** Primary and secondary antibodies used and their dilutions.

Antigen	Species	Dilution	Provider	Protocol Specifications
AIF-1	Goat polyclonal	1: 500	Abcam (ab5076)	EDTA pH = 9 before incubation with blocking solution
IL-12A	Rabbit monoclonal	1:100	Abcam (ab131039)	EDTA pH = 9 before incubation with blocking solution
IL-18	Rabbit monoclonal	1:250	Abcam (ab243091)	10 mM Sodium citrate pH = 6 before incubation with blocking solution
IL-10	Rabbit Polyclonal	1:100	Abcam (ab217941)	100% Triton 0.1% in PBS, 10 min, before incubation with blocking solution
IgG (Rabbit)	Mouse	1:1000	Sigma-Aldrich (RG-96/B5283)	------
IgG (Goat)	Mouse	1:100	Sigma-Aldrich [GT- 4/B3148]	------

## Data Availability

The data used to support the findings of the present study are available from the corresponding author upon request.

## References

[1] Youn Y.J., Lee J. (2019). Chronic venous insufficiency and varicose veins of the lower extremities. Korean J. Intern. Med..

[2] Raffetto J.D., Mannello F. (2014). Pathophysiology of chronic venous disease. Int. Angiol. Ed. Minerva Med..

[3] Barron G.S., Jacob S.E., Kirsner R.S. (2007). Dermatologic Complications of Chronic Venous Disease: Medical Management and Beyond. Ann. Vasc. Surg..

[4] Ortega M.A., Fraile-Martínez O., García-Montero C., Álvarez-Mon M.A., Chaowen C., Ruiz-Grande F., Pekarek L., Monserrat J., Asúnsolo A., García-Honduvilla N. (2021). Understanding Chronic Venous Disease: A Critical Overview of Its Pathophysiology and Medical Management. J. Clin. Med..

[5] Vlajinac H.D., Radak J., Marinković J.M., Maksimović M. (2012). Risk factors for chronic venous disease. Phlebology.

[6] de Barros N., Perez M.D.C.J., de Amorim J.E., Miranda F. (2010). Pregnancy and lower limb varicose veins: Prevalence and risk factors. J. Vasc. Bras. Soc. Bras. Angiol. Cir. Vasc. (SBACV).

[7] Ropacka-Lesiak M., Jarosław K., Bręborowicz G. (2015). Pregnancy-dependent blood flow velocity changes in lower extremities veins in venous insufficiency. Ginekol. Polska.

[8] Troiano N.H. (2018). Physiologic and hemodynamic changes during pregnancy. AACN Adv. Crit. Care Am. Assoc. Crit. Care Nurses.

[9] Labropoulos N. (2019). How Does Chronic Venous Disease Progress from the First Symptoms to the Advanced Stages? A Review. Adv. Ther. Springer Healthc..

[10] Taylor J., Hicks C., Heller J.A. (2018). The hemodynamic effects of pregnancy on the lower extremity venous system. J. Vasc. Surgery Venous Lymphat. Disord..

[11] Morton A. (2021). Physiological Changes and Cardiovascular Investigations in Pregnancy. Heart Lung Circ..

[12] Spence T., Allsopp P.J., Yeates A.J., Mulhern M.S., Strain J.J., McSorley E.M. (2021). Maternal Serum Cytokine Concentrations in Healthy Pregnancy and Preeclampsia. J. Pregnancy.

[13] Sykes L., MacIntyre D.A., Yap X.J., Teoh T.G., Bennett P.R. (2012). The Th1:Th2 dichotomy of pregnancy and preterm labour. Mediat. Inflamm..

[14] Ortega M.A., Gómez-Lahoz A.M., Sánchez-Trujillo L., Fraile-Martinez O., García-Montero C., Guijarro L.G., Bravo C., De Leon-Luis J.A., Saz J.V., Bujan J. (2022). Chronic Venous Disease during Pregnancy Causes a Systematic Increase in Maternal and Fetal Proinflammatory Markers. Int. J. Mol. Sci..

[15] Hammad I.A., Blue N., Allshouse A.A., Silver R.M., Gibbins K.J., Page J.M., Goldenberg R.L., Reddy U.M., Saade G.R., Dudley D.J. (2020). Umbilical Cord Abnormalities and Stillbirth. Obstet. Gynecol..

[16] Fajersztajn L., Veras M.M. (2017). Hypoxia: From Placental Development to Fetal Programming. Birth Defects Res..

[17] Konkel L. (2016). Lasting Impact of an Ephemeral Organ: The Role of the Placenta in Fetal Programming. Environ. Health Perspect..

[18] Ortega M.A., Saez M Á., Asúnsolo Á., Romero B., Bravo C., Coca S., Sainz F., Álvarez-Mon M., Buján J., García-Honduvilla N. (2019). Upregulation of VEGF and PEDF in Placentas of Women with Lower Extremity Venous Insufficiency during Pregnancy and Its Implication in Villous Calcification. Biomed. Res. Int..

[19] Ortega M.A., Romero B., Asúnsolo Á., Martínez-Vivero C., Sainz F., Bravo C., De León-Luis J., Álvarez-Mon M., Buján J., García-Honduvilla N. (2020). Pregnancy-associated venous insufficiency course with placental and systemic oxidative stress. J. Cell. Mol. Med..

[20] Ortega M.A., Saez M.A., Fraile-Martínez O., Asúnsolo Á., Pekarek L., Bravo C., Coca S., Sainz F., Mon M.Á., Buján J. (2020). Increased Angiogenesis and Lymphangiogenesis in the Placental Villi of Women with Chronic Venous Disease during Pregnancy. Int. J. Mol. Sci..

[21] Honduvilla N.G., A Ortega M., Asúnsolo Á., Álvarez-Rocha M.J., Romero B., De León-Luis J., Álvarez-Mon M., Buján J. (2018). Placentas from women with pregnancy-associated venous insufficiency show villi damage with evidence of hypoxic cellular stress. Hum. Pathol..

[22] Ortega M.A., Sánchez-Trujillo L., Bravo C., Fraile-Martinez O., García-Montero C., Saez M.A., Alvarez-Mon M.A., Sainz F., Alvarez-Mon M., Bujan J. (2021). Newborns of Mothers with Venous Disease during Pregnancy Show Increased Levels of Lipid Peroxidation and Markers of Oxidative Stress and Hypoxia in the Umbilical Cord. Antioxidants.

[23] Fragoso M.B.T., Ferreira R.C., Tenório M.C.D.S., Moura F.A., de Araújo O.R.P., Bueno N.B., Goulart M.O.F., de Oliveira A.C.M. (2021). Biomarkers of Inflammation and Redox Imbalance in Umbilical Cord in Pregnancies with and without Preeclampsia and Consequent Perinatal Outcomes. Oxidative Med. Cell. Longev..

[24] Oh J.-W., Park C.-W., Moon K.C., Park J.S., Jun J.K. (2019). The relationship among the progression of inflammation in umbilical cord, fetal inflammatory response, early-onset neonatal sepsis, and chorioamnionitis. PLoS ONE.

[25] Redline R.W. (2006). Inflammatory responses in the placenta and umbilical cord. Semin. Fetal Neonatal Med..

[26] Deininger M.H., Meyermann R., Schluesener H.J. (2002). The allograft inflammatory factor-1 family of proteins. FEBS Lett..

[27] Sikora M., Kopeć B., Piotrowska K., Pawlik A. (2019). Role of allograft inflammatory factor-1 in pathogenesis of diseases. Immunol. Lett..

[28] Lurie F., Passman M., Meisner M., Dalsing M., Masuda E., Welch H., Bush R.L., Blebea J., Carpentier P.H., De Maeseneer M. (2020). The 2020 update of the CEAP classification system and reporting standards. J. Vasc. Surg. Venous Lymphat. Disord..

[29] Ortega M.A., Chaowen C., Fraile-Martinez O., García-Montero C., Saez M.A., Cruza I., Pereda-Cerquella C., Alvarez-Mon M.A., Guijarro L.G., Fatych Y. (2022). Chronic Venous Disease in Pregnant Women Causes an Increase in ILK in the Placental Villi Associated with a Decrease in E-Cadherin. J. Pers. Med..

[30] Guss L.G., Javvaji S., Case J., Bs B.B., Schaefer K.N., Bs R.G., Waalen J., Greenway H.T., Housman L.B. (2018). Differences in Inflammatory Cytokine Levels between Patients with Varying Severity of Chronic Venous Insufficiency. J. Vasc. Med. Surg..

[31] Ortega M.A., Fraile-Martínez O., Saez M.A., Álvarez-Mon M.A., Gómez-Lahoz A.M., Bravo C., Luis J.A.L., Sainz F., Coca S., Asúnsolo Á. (2021). Abnormal proinflammatory and stressor environmental with increased the regulatory cellular IGF-1/PAPP-A/STC and Wnt-1/β-Catenin canonical pathway in placenta of women with Chronic venous Disease during Pregnancy. Int. J. Med. Sci..

[32] Ortega M.A., Romero B., Asúnsolo Á., Sainz F., Martinez-Vivero C., Álvarez-Mon M., Buján J., García-Honduvilla N. (2018). Behavior of Smooth Muscle Cells under Hypoxic Conditions: Possible Implications on the Varicose Vein Endothelium. BioMed Res. Int..

[33] Lohr J.M., Bush R.L. (2013). Venous disease in women: Epidemiology, manifestations, and treatment. J. Vasc. Surg..

[34] Asúnsolo Á., Chaowen C., Ortega M.A., Coca S., Borrell L.N., De León-Luis J., García-Honduvilla N., Álvarez-Mon M., Buján J. (2021). Association Between Lower Extremity Venous Insufficiency and Intrapartum Fetal Compromise: A Nationwide Cross-Sectional Study. Front. Med..

[35] De Leon-Oliva D., Garcia-Montero C., Fraile-Martinez O., Boaru D.L., García-Puente L., Rios-Parra A., Garrido-Gil M.J., Casanova-Martín C., García-Honduvilla N., Bujan J. (2023). AIF1: Function and Connection with Inflammatory Diseases. Biology.

[36] Zhao Y.-Y., Yan D.-J., Chen Z.-W. (2013). Role of AIF-1 in the regulation of inflammatory activation and diverse disease processes. Cell. Immunol..

[37] Jia J., Cai Y., Wang R., Fu K., Zhao Y.-F. (2010). Overexpression of Allograft Inflammatory Factor-1 Promotes the Proliferation and Migration of Human Endothelial Cells (HUV-EC-C) Probably by Up-Regulation of Basic Fibroblast Growth Factor. Pediatr. Res..

[38] Ortega M.A., Romero B., Asúnsolo Á., Sola M., Álavrez-Rocha M.J., Sainz F., Álavrez-Mon M., Buján J., García-Honduvilla N. (2019). Patients with Incompetent Valves in Chronic Venous Insufficiency Show Increased Systematic Lipid Peroxidation and Cellular Oxidative Stress Markers. Oxid. Med. Cell. Longev..

[39] Yang Z.F., Ho D.W., Lau C.K., Lam C.T., Lum C.T., Poon R.T.P., Fan S.T. (2005). Allograft inflammatory factor-1 (AIF-1) is crucial for the survival and pro-inflammatory activity of macrophages. Int. Immunol..

[40] Liu G., Ma H., Jiang L., Zhao Y. (2007). Allograft inflammatory factor-1 and its immune regulation. Autoimmunity.

[41] Kadoya M., Yamamoto A., Hamaguchi M., Obayashi H., Mizushima K., Ohta M., Seno T., Oda R., Fujiwara H., Kohno M. (2014). Allograft inflammatory factor-1 stimulates chemokine production and induces chemotaxis in human peripheral blood mononuclear cells. Biochem. Biophys. Res. Commun..

[42] Behzadi P., Behzadi E., Ranjbar R. (2016). IL-12 Family Cytokines: General Characteristics, Pathogenic Microorganisms, Receptors, and Signalling Pathways. Acta Microbiol. Immunol. Hung..

[43] Vignali D.A.A., Kuchroo V.K. (2012). IL-12 family cytokines: Immunological playmakers. Nat. Immunol..

[44] Becker C., Wirtz S., Neurath M.F. (2005). Stepwise Regulation of T H 1 Responses in Autoimmunity: IL-12-related Cytokines and Their Receptors IL-12 Receptor Signaling. Inflamm. Bowel Dis..

[45] Perricone C., De Carolis C., Perricone R. (2012). Pregnancy and autoimmunity: A common problem. Best. Pract. Res. Clin. Rheumatol..

[46] El-Kabarity R.H., Naguib A.H. (2011). Serum levels of IL-18, IL-12 and TH-1/TH-2 ratio in patients with pre-eclampsia. Egypt. J. Immunol..

[47] Yasuda K., Nakanishi K., Tsutsui H. (2019). Interleukin-18 in Health and Disease. Int. J. Mol. Sci..

[48] Tsutsui H., Nakanishi K. (2012). Immunotherapeutic applications of IL-18. Immunotherapy.

[49] Wawrocki S., Druszczynska M., Kowalewicz-Kulbat M., Rudnicka W. (2016). Interleukin 18 (IL-18) as a target for immune intervention. Acta Biochim. Pol..

[50] van de Veerdonk F.L., Netea M.G., Dinarello C.A., Joosten L.A. (2011). Inflammasome activation and IL-1&β; and IL-18 processing during infection. Trends Immunol..

[51] Menon R., Lombardi S.J., Fortunato S.J. (2001). IL-18, a Product of Choriodecidual Cells, Increases During Premature Rupture of Membranes but Fails to Turn on the Fas-FasL-Mediated Apoptosis Pathway. J. Assist. Reprod. Genet..

[52] Naeimi S., Ghiam A.F., Mojtahedi Z., Dehaghani A.S., Amani D., Ghaderi A. (2006). Interleukin-18 gene promoter polymorphisms and recurrent spontaneous abortion. Eur. J. Obstet. Gynecol. Reprod. Biol..

[53] Huang X., Huang H., Dong M., Yao Q., Wang H. (2005). Serum and placental interleukin-18 are elevated in preeclampsia. J. Reprod. Immunol..

[54] Saraiva M., O’Garra A. (2010). The regulation of IL-10 production by immune cells. Nat. Rev. Immunol..

[55] Wang X., Wong K., Ouyang W., Rutz S. (2017). Targeting IL-10 Family Cytokines for the Treatment of Human Diseases. Cold Spring Harb. Perspect. Biol..

[56] Justiz Vaillant A.A., Qurie A. (2021). Interleukin. StatPearls.

[57] Brogin Moreli J., Cirino Ruocco A.M., Vernini J.M., Rudge M.V.C., Calderon I.M.P. (2012). Interleukin 10 and Tumor Necrosis Factor-Alpha in Pregnancy: Aspects of Interest in Clinical Obstetrics. ISRN Obstet. Gynecol..

[58] Chatterjee P., Chiasson V.L., Bounds K.R., Mitchell B.M. (2014). Regulation of the anti-inflammatory cytokines interleukin-4 and interleukin-10 during pregnancy. Front. Immunol..

[59] Méndez-García L.A., Minor-Borrego H., Sánchez-Del Real A.L., Aguayo-Guerrero J.A., Alvarado-Monroy T., Trejo-Millán F., Rosas-Salinas J., Rizo-Tellez S.A., Islas-Andrade S., Briones-Garduño J.C. (2021). Cord blood levels of interleukin-10 decrease in neonates with increased birth weight: Novel implications of the cytokine network in early obesity. Eur. J. Pediatr..

[60] Rocha G., Proença E., Guedes A., Carvalho C., Areias A., Ramos J.P., Rodrigues T., Guimarães H. (2012). Cord blood levels of IL-6, IL-8 and IL-10 may be early predictors of bronchopulmonary dysplasia in preterm newborns small for gestational age. Dis. Markers.

